# Association between dimensions of trauma-related psychopathology and asthma in trauma-exposed women

**DOI:** 10.3389/fnbeh.2023.1268877

**Published:** 2023-10-31

**Authors:** Esther R.-H. Lin, Alyssa R. Roeckner, Negar Fani, Natalie Merrill, Charles F. Gillespie, Timothy D. Ely, Bekh Bradley, Vasiliki Michopoulos, Abigail Powers, Tanja Jovanovic, Jennifer S. Stevens

**Affiliations:** ^1^Department of Psychiatry and Behavioral Sciences, Emory University School of Medicine, Atlanta, GA, United States; ^2^Atlanta VA Health Care System, Atlanta, GA, United States; ^3^Wayne State University School of Medicine, Detroit, MI, United States

**Keywords:** fMRI, asthma, PTSD, women’s health, community sample

## Abstract

**Introduction:**

Exposure to traumatic events and stressful life experiences are associated with a wide range of adverse mental and physical health outcomes. Studies have found post-traumatic stress disorder (PTSD), depression, and anxiety sensitivity occurrence to be common in addition to inflammatory diseases like asthma, especially in women. Moreover, overlapping neurobiological mechanisms have been linked to both PTSD and asthma.

**Methods:**

In the current study, n = 508 women reported on presence of lifetime asthma diagnosis and symptoms of trauma-related psychopathology including PTSD and depression. A separate group of female participants (n = 64) reported on asthma, PTSD, depression and anxiety sensitivity, and underwent functional MRI scans during a fearful faces task, and their anterior insula responses were analyzed.

**Results:**

Overall, PTSD and depression severity were significantly higher in those with asthma versus those without asthma. There was a positive association between anterior insula response to social threat cues and depression symptoms only among individuals without a lifetime presence of asthma.

**Discussion:**

These findings provide continued evidence on the interactions between stress, neural mechanisms involved in interoception and salience detection, and trauma-related psychopathology.

## 1. Introduction

Lifetime stress and trauma exposure can be moderating factors for disease vulnerability and severity. In response to stressors, individuals may develop emotional distress that can result in disorders like PTSD and depression, or intense chronic stress symptoms including anxiety sensitivity (Breslau et al., 1991; Holman et al., 2000; Yehuda et al., 2001; Rayburn et al., 2005; Benjet et al., 2016; Gluck et al., 2021). Stress and trauma are also associated with greater risk for physical diseases as studies have reported physiological changes and disease development in humans following exposure to traumatic events including inflammatory diseases such as asthma (Chung et al., 2012; Alonso et al., 2014; Ohno, 2017; Hung et al., 2019; Allgire et al., 2021). A disparate burden for PTSD, depression, anxiety, and asthma exists among women compared to men as women are at higher risk for developing these disorders and experiencing greater symptom severity (Breslau et al., 1997; Lang et al., 2002; Rayburn et al., 2005; Alim et al., 2006; Chowdhury et al., 2021). Previous studies identified that the prevalence of lifetime PTSD, depression, and anxiety symptoms is higher in trauma-exposed women, especially those living in urban areas where asthma comorbidity is also greater (Lang et al., 2002; Alim et al., 2006; Li et al., 2013; Lawson et al., 2017; Rodriguez et al., 2019). Understanding the overlap between psychological disorders and somatic diseases in trauma-exposed women may contribute to the development of personalized treatments to improve health outcomes for those most at risk. Exposure to traumatic events is a prerequisite to diagnosing PTSD, a trauma-induced psychiatric condition that affects millions of adults in a given year, causing symptoms categorized into clusters including reexperiencing, intrusion, avoidance and numbing, and hyperarousal (Pacella et al., 2013; Wendlandt et al., 2022). Stress and trauma are also potent risk factors for developing depression (Breslau et al., 2000; O’Donnell et al., 2004; Heim et al., 2008; Elhai et al., 2011; Fowler et al., 2013; Cattaneo et al., 2015). Almost one in ten Americans have some type of depression, with symptoms such as trouble sleeping or being unable to feel pleasure or interest in things one used to enjoy. Trauma cues and stressful experiences are also associated with anxiety sensitivity, the tendency to fear anxiety-related sensations and symptoms of anxiety (McNally, 2002; Vujanovic et al., 2012). Higher rates of anxiety sensitivity are found among individuals suffering from elevated levels of stressful and traumatic events, as studies have consistently found participants with a trauma history to score higher on anxiety sensitivity measures (Lang et al., 2002; Feldner et al., 2006; Asmundson and Stapleton, 2008; Vujanovic et al., 2012; McHugh et al., 2017; Paulus et al., 2018; Zvolensky et al., 2020). Together, stress and trauma exposure appear to exacerbate symptomatology outcomes.

These negative health outcomes can cause lasting dysfunction in daily life, not only from the stress- and trauma-induced mental distress, but also from physical diseases associated with it. There is compelling evidence that stressful and traumatic life events are associated with alterations in the magnitude of airway inflammatory responses in asthmatic individuals, and in increasing the risk for symptoms of PTSD and depression (Chen and Miller, 2007; Van Lieshout and MacQueen, 2008; Ohno, 2010; Trueba and Ritz, 2013; Miyasaka et al., 2018). Individuals who experience major stressors are more vulnerable to immune dysregulation and several lines of evidence link PTSD, major depression, and anxiety sensitivity to asthma. Psychological stress may increase the frequency and severity of asthmatics’ symptoms and enhance airway inflammatory responses seen by an increase in levels of proinflammatory mediators (Allgire et al., 2021). Studies involving participants worldwide and in hospitals nationwide found that PTSD and stress symptoms occur more commonly in people with asthma than expected and are associated with poorer symptom outcomes (Kewalramani et al., 2008; Chung et al., 2012; Alonso et al., 2014). A strong association between asthma development and depression severity has also been observed in diverse populations nationwide and in longitudinal studies (Goodwin et al., 2004; Eisner et al., 2005; Trojan et al., 2014; Akula et al., 2018; Choi et al., 2019). Furthermore, greater anxiety sensitivity levels have consistently been reported in asthmatics and in predicting asthma symptom severity (Barone et al., 2008; McLeish et al., 2011, 2016; Avallone et al., 2012). Like PTSD, depression, and anxiety sensitivity, asthma is also more prevalent in women than men as epidemiological studies have reported a higher preponderance of asthma in women after age thirteen or the onset of puberty (Chowdhury et al., 2021). These robust findings on comorbid asthma and psychological disorder presentations suggest the possibility of underlying mechanisms and factors contributing to a shared pathophysiology between stress and asthma, which may include brain function.

Recent findings from prospective studies of stress and asthma implicate the anterior insula as a potential overlapping mediator between somatic diseases and psychiatric disorders. The anterior insula is a primary neural substrate critically involved in processing emotions, interoceptive information, and somatic sensations (Fonzo et al., 2010; Uddin et al., 2017; Park et al., 2022). Relative to other brain regions, there is altered insula activation in response to threat cues, emotional face stimuli, and paradigms on intero−/exteroceptive awareness in those with PTSD, depression, anxiety sensitivity, and asthma, relative to healthy controls (Fonzo et al., 2010; Bruce et al., 2013; Duval et al., 2020). Specifically, patients with PTSD and women exposed to domestic violence show increased activity in the anterior insula (Simmons et al., 2008; Bruce et al., 2013). The anterior insula response to threat cues has also been suggested to be associated with PTSD symptoms of hyperarousal and re-experiencing (Yehuda et al., 2015; Stevens et al., 2018; Leroy et al., 2022). Though limited research has been done on anxiety sensitivity and anterior insula reactivity, a few studies have found anxiety sensitivity scores to be positively correlated with anterior insula activation in adults (Stein et al., 2007; Killgore et al., 2011; Yang et al., 2016). Studies on patients with major depressive disorder have yielded mixed findings, as some found anterior insula activation inversely correlated with depression severity while others found greater insula activation in depressed participants compared to healthy controls (Strigo et al., 2010; Wiebking et al., 2010; Perlman et al., 2012; Avery et al., 2014; McIntosh et al., 2022). Together, these findings suggest some differences in insula function in PTSD versus depression, although a number of studies of depression lack information on participants’ trauma exposure.

There is also evidence of alterations in anterior insula activation among individuals with asthma. Researchers have found the degree of differential insula activation to predict changes in airway inflammation. Specifically, individuals with greater insula responses to asthma-specific emotional cues following allergen exposure, had greater inflammatory signals in their lungs and greater severity of the disease (Rosenkranz et al., 2012). Though the link between asthma and the insula remains an emerging research topic, studies of inflammation and the insula have theorized that inflammation may induce increased insula sensitivity to interoceptive signals (Karshikoff et al., 2017). Taking these findings together along with the insula’s crucial role in serving as an interface between external stimuli and stimuli originating inside the body (Wiebking et al., 2015), the anterior insula appears to be a promising area of interest in the relation between stress, psychopathology, and asthma.

In the present study, we investigated the relationship between asthma and PTSD, depression, and anxiety sensitivity symptoms using a large sample of African American women in a highly trauma-exposed, low socioeconomic status urban hospital setting. In another sample who completed functional magnetic resonance imaging (fMRI), we also investigated asthma and PTSD, depression, and anxiety sensitivity association in relation to anterior insula reactivity to social threat cues. In line with previous research, we posit that asthma contributes to the development of symptoms of trauma-related psychopathologies such that experiencing more stressful or traumatic events may accentuate greater airway inflammatory responses and, in doing so, increases the likelihood of developing symptoms (Chen and Miller, 2007). The increase in psychological stress symptoms and suppressed immune responses could be reflected through altered insula responses. Based on prior findings regarding stress, asthma, and psychopathology (Chen and Miller, 2007; McLeish et al., 2016; Hung et al., 2019; Allgire et al., 2021), we hypothesized that women with asthma would have greater PTSD severity, depression severity, and anxiety sensitivity symptoms than women without asthma. Secondly, building upon previous research positing the insula’s involvement in asthma and PTSD, anxiety sensitivity, and depression (Killgore et al., 2011; Bruce et al., 2013; Avery et al., 2014), we hypothesized that greater anterior insula reactivity to social threat cues would moderate associations between asthma and PTSD, depression, and anxiety sensitivity symptoms severity.

## 2. Methods

### 2.1. Participants

Participants included women between the ages of 18 and 65 recruited from a larger ongoing study of risk factors for PTSD in the Grady Trauma Project. Participants were randomly approached in the outpatient clinics and the emergency room of Grady Memorial Hospital, a public inner-city hospital that serves a primarily low-income, African American population in Atlanta, Georgia. High rates of trauma and posttraumatic symptoms have been previously observed within this patient population (Gillespie et al., 2009; Gluck et al., 2021). For the MRI sample, participants were excluded if they met any of the following exclusion criteria based on self-report: history of neurological disorder, psychosis, or current psychotropic medication, metal clips or implants, history of head injury or loss of consciousness exceeding 5 min. As well, participants who tested positive for pregnancy or illegal drug use based on urine tests 24 h before the MRI scan were excluded. Participants were English-speaking and provided written informed consent prior to the study, and compensation was provided. The Institutional Review Board of Emory University and the Research Oversight Committee of Grady Memorial Hospital approved all study procedures in accordance with the Declaration of Helsinki.

Female participants (*n* = 508) in the Grady Trauma Project reported on asthma presence, with *n* = 161 (31.69%) reporting a diagnosis of asthma (Table 1). Among these, *n* = 242 met criteria for PTSD diagnosis. The fMRI analysis included a sample of *n* = 64 Black or African American women, among whom *n* = 10 were diagnosed with asthma (Table 2). In this sample, *n* = 27 met criteria for PTSD diagnosis. In the primary sample, asthma was self-reported during screening. In the MRI study, asthma was reported in a physical health and medical history assessment conducted by a practicing physician.

**Table 1:** Clinical and demographic features of general sample.

Demographics	Asthma	No Asthma
	(*n* = 161)	(*n* = 347)
Age, mean (SD)	41.43 (13.46)	40.07 (13.65)
PCL-5, mean (SD)	34.28 (21.21)	28.45 (20.08)
BDI, mean (SD)	23.24 (14.30)	19.31 (13.32)
CTQ, mean (SD)	53.32 (21.22)	49.73 (21.14)
TEI, mean (SD)	6.21 (3.57)	5.18 (3.06)
Asthma medication (%)		
Yes	101 (62.73%)	N/A
No	60 (37.28%)	N/A
BMI, mean (SD)	33.85 (9.52)	30.57 (7.85)
Smoking Tobacco (%)		
Yes	52 (32.30%)	101 (29.11%)
No	109 (67.70%)	246 (70.89%)
Race (%)		
African American/Black	148 (91.93%)	308 (88.76%)
Hispanic/Latino	0 (0.00%)	6 (1.73%)
Asian	0 (0.00%)	0 (0.00%)
Caucasian/White	7 (4.35%)	18 (5.19%)
Mixed	3 (1.86%)	12 (3.46%)
Other	5 (3.11%)	1 (0.29%)
Education (%)		
Some high school	31 (19.25%)	41 (11.82%)
High school degree	35 (21.74%)	91 (26.22%)
GED	5 (3.11%)	13 (3.75%)
Some college or technical school	44 (27.33%)	95 (27.38%)
Technical school graduate	15 (9.32%)	22 (6.34%)
College graduate	21 (13.04%)	61 (17.58%)
Graduate school	11 (6.83%)	24 (6.92%)
Relationship status (%)		
Single/Never married	94 (58.39%)	216 (62.25%)
Married	21 (13.04%)	39 (11.24%)
Divorced	23 (14.29%)	46 (13.26%)
Separated	8 (4.97%)	14 (4.03%)
Widowed	11 (6.83%)	12 (3.46%)
Domestic partner	5 (3.11%)	18 (5.19%)

PTSD-Checklist DSM-5 (PCL-5) Range: 0–80, Beck Depression Inventory (BDI) Range: 0–63, Childhood Trauma Questionnaire (CTQ) Range: 5–125. Traumatic Events Inventory (TEI).

**Table 2:** Clinical and demographic features of fMRI sample.

Demographics	Asthma	No Asthma
	(*n* = 10)	(*n* = 54)
Age, mean (SD)	36.40 (10.81)	39.61 (11.51)
PSS, mean (SD)	13.80 (12.12)	11.81 (11.61)
TEI, mean (SD)	4.16 (1.77)	4.65 (2.36)
ASI, mean (SD)	24.70 (16.02)	26.00 (14.67)
BDI, mean (SD)	13.40 (12.61)	13.58 (12.97)
CTQ, mean (SD)	47.70 (20.92)	39.46 (17.14)
Asthma medication (%)		
Yes	5 (50.00%)	N/A
No	5 (50.00%)	N/A
BMI, mean (SD)	34.33 (5.16)	31.76 (6.44)
Education (%)		
Some high school	1 (10.00%)	11 (20.37%)
High school degree	7 (70.00%)	9 (16.67%)
GED	1 (10.00%)	3 (5.56%)
Some college or technical school	0 (0%)	16 (29.63%)
Technical school graduate	1 (10.00%)	7 (12.96%)
College graduate	0 (0%)	5 (9.26%)
Graduate school	0 (0%)	3 (5.56%)

PTSD Symptom Scale (PSS) Range: 0–40, Traumatic Events Inventory (TEI), Anxiety Sensitivity Index (ASI) Range: 0–64, Beck Depression Inventory (BDI) Range: 0–63.

### 2.2. Measures

Following enrollment, trained interviewers collected detailed demographic and health information and administered an extensive array of psychological measures during a two-hour in-person screen in the laboratory. The study included demographic information on age, sex, race, ethnicity, education, and household monthly income. Since asthma is a primary variable of interest, asthma presence was assessed by the question “Have you ever been diagnosed with asthma?” with answer choices as either “yes” or “no.” For the neuroimaging analysis, asthma diagnosis was ascertained from participant medical history.

Various psychological assessments were administered during the initial participant screen. The focus of the present study are data from the following:

#### 2.2.1. PTSD Checklist for DSM-5

The PTSD Checklist for DSM-5 (PCL-5; Weathers et al., 2013) was used to assess the 20 DSM-5 symptoms of PTSD. The PCL-5 has a variety of purposes including monitoring symptom change during and after treatment, screening individuals for PTSD, and making a provisional PTSD diagnosis. For the purposes of this study, the PCL-5 was used to screen individuals for PTSD. Specifically, the PCL-5 provided quantitative data on the participant’s PTSD severity. The PCL-5 is a 20-item self-report measure corresponding to the DSM-5 symptom criteria for PTSD. A sample item includes: “In the past month, how much were you bothered by repeated, disturbing, and unwanted memories of the stressful experience” or “In the past month, how much were you bothered by avoiding external reminders of the stressful experience (for example, people, places, conversations, activities, objects, or situations)?.” The self-report rating scale is based on a 5-point Likert scale (0 = “Not at all,” 1 = “A little bit,” 2 = “Moderately,” 3 = “Quite a bit,” and 4 = “Extremely”). A total symptom severity score (0–80) can be obtained by summing the scores for each of the 20 items. We implemented a PCL-5 cutoff score of 31 as indicative of probable PTSD. The PCL-5 total score was used to index PTSD symptom severity in the primary sample.

#### 2.2.2. PTSD Symptom Scale

The PSS was the primary measure to assess PTSD severity in the neuroimaging group of participants. The PTSD Symptom Scale (PSS; Foa and Tolin, 2000) was used to assess the frequency and severity of 17 DSM-IV-TR symptoms of PTSD. The PSS is a 17-item self-report measure that asks participants to rate the frequency of symptoms experienced over the past 2 weeks, from 0 (“Not at all/only once”) to 3 (“Almost always/5 or more times per week”). Symptoms are divided into three clusters corresponding to DSM-IV-TR criteria: re-experiencing (i.e., flashbacks/trauma-related nightmares), avoidance (i.e., avoiding certain people, places, situations related to the event), and hyperarousal (i.e., jumpiness, easily startled). A final question asks participants to report the length of their symptoms, from 0 (“less than a month”) to 3 (“greater than 1 year”). The total score is calculated by averaging individual items and multiplying the average by 17, yielding a maximum score of 51.

#### 2.2.3. Beck Depression Inventory

The Beck Depression Inventory (BDI; Beck et al., 1996) is a widely used and well-validated 21-item self-report inventory that measures the severity and symptoms of depression. Participants are asked to rate each item the extent to which they feel the statements describe how they have been feeling during the past 2 weeks on a 4-point scale, from 0 (“I get as much pleasure as I ever did from the things I enjoy”) to 3 (“I cannot get any pleasure from the things I used to enjoy”). The scores were totaled and depression symptom severity was considered minimal (scores 0 to 9), mild (10 to 18), moderate (19 to 29), or severe (30 to 63).

#### 2.2.4. Anxiety Sensitivity Index

The Anxiety Sensitivity Index (ASI; Reiss et al., 1986) is a 16-item scale used to measure fear of anxiety-related sensations that individuals could have, particularly those common in panic disorder. A higher score on the ASI serves as a powerful and unique predictor of people with a high risk of anxiety disorders such as panic disorders and phobias or as an indicator of post-traumatic stress. The ASI asks participants to rate each item on a 5-point scale, from 0 (*very little*) to 4 (*very much*). An individual’s score is the sum of the scores on the 16 items.

#### 2.2.5. Childhood Trauma Questionnaire

The Childhood Trauma Questionnaire (CTQ; Bernstein and Fink, 1998) is a self-report inventory that measures five categories of traumatic experiences in childhood: physical abuse, physical neglect, emotional abuse, emotional neglect, and sexual abuse, that occurred between birth until the age of 17 years. This 28-item screen asks participants to rank the extent to which they believe the statements to be true about their experiences as a child and teenager, from 1 (“never true”) to 5 (“always true”). The measure yields a total score for childhood trauma as well as subscores for each of the five categories of maltreatment, with higher scores indicative of more abuse. In a prior study with this sample, Cronbach’s *a* was 0.83 for physical abuse, 0.86 for emotional abuse, and 0.95 for sexual abuse (Mandavia et al., 2016).

#### 2.2.6. Traumatic Events Inventory

The Traumatic Events Inventory (TEI; Schwartz et al., 2005) is a 14-item screen for assessing lifetime trauma history in individuals. The TEI asks about any traumatic or stressful events that the participants may have experienced, witnessed, or been confronted with in their lifetime, as well as the age of first exposure and frequency of exposure. Trauma types queried include natural disasters, serious accidents or injuries, military combat, being confronted with the murder of a close friend or family member, being attacked with and without a weapon, witnessing violence between parents or caregivers as a child, being beaten or physically punished, verbally abused, or sexually abused as a child, and being raped or sexually assaulted as an adult. An open-ended question also asks about any other events that may have been traumatic or particularly stressful for the participant. The total number of trauma types experienced or witnessed during participants’ childhood and adulthood was used to further identify any relation between traumatic events and asthma (Mekawi et al., 2021).

### 2.3. Brain imaging procedures

A sub-sample of female participants (*n* = 64) completed an fMRI scan while viewing the fearful faces task. The fMRI study procedures have been published previously and followed Stevens et al. (2013). Eight fearful and eight neutral (4 male and 4 female) faces were selected from a stimulus set of Ekman and Friesen (1976). Stimuli were projected onto a 24-inch screen at a resolution of 1,280 × 1,024 using EPrime 2.0 software (Psychology Software Tools, Pittsburgh, PA). Participants viewed 30 blocks of static fearful and neutral face stimuli of Caucasian race (15 fearful and 15 neutral blocks that were randomly intermixed). Each block consisted of eight faces presented in a random order. Each individual face stimuli were presented for 500 ms, followed by a 500 ms presentation of a fixation cross. A 10,000 ms rest period was presented after every 10th block during which participants were instructed to “relax and look at the screen.” Face stimuli were presented at a size of 4.3 × 6.7′ on a black background, and the fixation cross and instructions were presented in white 18-point Courier New font on a black background.

### 2.4. Brain imaging acquisition and analysis

Brain imaging data were acquired on a Siemens 3.0 T Magnetom Trio TIM MRI Scanner using a 12-channel coil. Data acquisitions and preprocessing followed methods outlined in Kilaru et al. (2010). Correction for slice timing and spatial realignment were applied to images in SPM8. Images were then normalized with unified segmentation and smoothed with a 9 mm Gaussian kernel. Anterior insula ROI was created using a 10 mm sphere based on coordinates defined from a prior PTSD neuroimaging study (Laird et al., 2011; Leroy et al., 2022).

### 2.5. Statistical analyses

All statistical analyses were run in R version 4.2.1 and R Studio version 2022.07.2. The dplyr package in R was utilized for data organization. Statistical significance was set at *α* = 0.05 (2-tailed) for all analyses.

In order to assess the association between asthma and PTSD severity, an independent sample, two-tailed Welsch’s t-test was used. This tested group differences in PTSD severity in women with vs. without asthma. The same analyses were used to test for asthma and depression severity, and asthma and anxiety sensitivity severity. All comparisons of the asthma and no asthma groups controlled for age as a covariate. Secondary sensitivity analyses tested whether any significant findings held after controlling for body mass index (BMI), lifetime trauma load (TEI), and childhood maltreatment severity (CTQ).

To investigate how the association between asthma and PTSD relates to anterior insula activity in women, regression models were used to test for asthma*anterior insula interaction effects on PTSD symptoms collected at the time of scan, as measured by the PSS. Age was included as a covariate. For any significant findings, we tested whether the findings would hold after controlling for BMI and asthma medication usage as covariates. Follow-up analyses to test for asthma*anterior insula interaction effects on BDI and ASI were also conducted.

Exploratory whole-brain, multiple regression analyses were conducted with asthma and PSS scores as separate covariates and as an interaction effect (asthma*PSS). The same analyses were completed for asthma and ASI as well as asthma and BDI. The resulting maps were tested for significance using cluster-defining threshold of *p* < 0.005, with cluster-level family-wise error correction set to *p* < 0.05. This produced an extent threshold of *k* = 212 for PSS score correlations, *k* = 125 for ASI score correlations, and *k* = 219 for BDI score correlations.

## 3. Results

### 3.1. Sample characteristics

Table 1 lists the clinical and demographic characteristics of the primary sample. Within the asthma group (*n* = 161; 31.69%), most participants reported taking asthma medication (*n* = 101 or 62.73%). Table 2 shows the clinical and demographic features of the sample who completed neuroimaging. Within the asthma group (*n* = 10; 15.63%), all participants completed a physical and medical history exam with a physician on the study, prior to the MRI scan. During this exam, half of the participants (*n* = 5; 50.00%) reported taking asthma medication including albuterol, levalbuterol, budesonide-formoterol, and loratadine during their lifetime. Participants were asked to abstain from any non-medically necessary drugs on the day of the scan.

### 3.2. Differences in PTSD severity among women with asthma vs. without asthma

**Figure 1:**
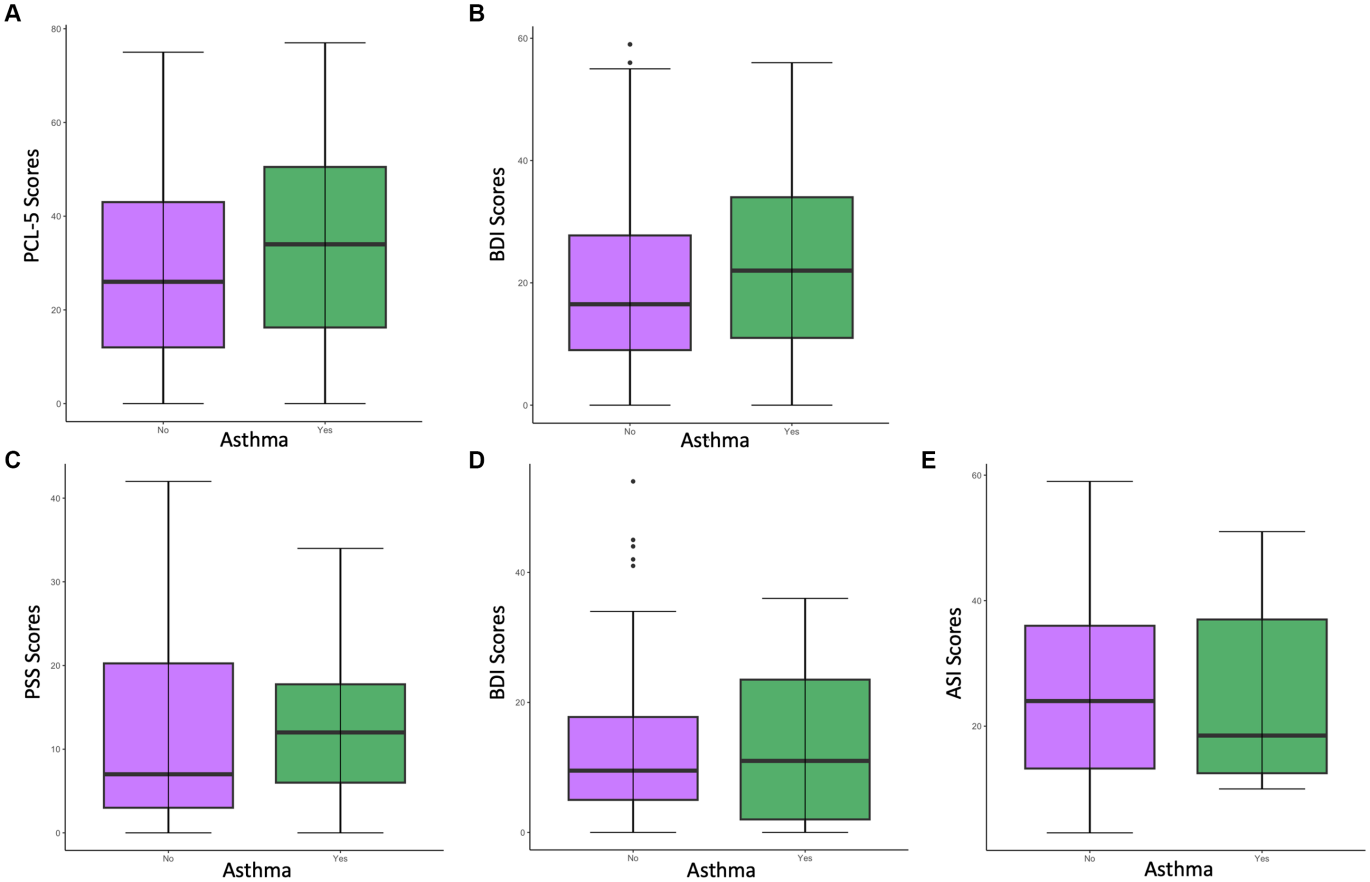
Differences in psychological measure scores among women with asthma vs. without asthma in the primary sample **(A,B)** and the neuroimaging sample **(C–E)**. **(A)** Differences in PCL-5 scores among women with vs. without asthma in the primary sample. Results indicated a significant difference (*p* < 0.05) in PCL-5 scores between women with vs. without asthma, with the asthma group having significantly higher PCL-5 scores (*M* = 34.28). **(B)** Differences in BDI scores indicated a significant difference (*p* < 0.005) in BDI scores among women with vs. without asthma, with the asthma group having significantly higher BDI scores (*M* = 23.24). **(C)** Differences in PSS scores among women with vs. without asthma in the neuroimaging sample. **(D)** Differences in BDI scores among women with vs. without asthma in the neuroimaging sample. **(E)** Differences in ASI scores among women with vs. without asthma in the neuroimaging sample.

First, we examined whether women with asthma have higher PTSD symptom severity than women without asthma in the primary sample. As shown in Figure 1A, women with asthma (*M* = 34.28, *SD* = 21.21) had significantly higher PCL-5 scores than those without asthma (*M* = 28.45, *SD* = 20.08), *t* = −3.01, *p* = 0.003. Participants with asthma also endorsed higher levels of childhood maltreatment (CTQ total *M* = 53.32, *SD* = 21.22), reported more traumatic events in their lifetime (TEI total *M* = 6.20, *SD* = 3.36), and had a higher body-mass-index (BMI *M* = 33.85, *SD* = 9.52) than participants without asthma. There was still a significant effect of asthma on PCL-5 scores after controlling for the effect of the CTQ, *F*(1, 500) = 6.35*, p* = 0.01, and BMI, *F*(1,505) = 7.71, *p* = 0.006, but not when controlling for the effect of the TEI, *F*(1, 502) = 2.54, *p* = 0.11. Both the CTQ and TEI were positively related to the participants’ PCL-5 scores [CTQ: *r*(500) = 0.51, *p* < 0.001; TEI: *r*(502) = 0.53, *p* < 0.001], but BMI was not [BMI: *r*(505) = 0.07, *p* = 0.10].

We also examined whether women with asthma have greater depression severity than women without asthma in the general sample. As shown in Figure 1B, women with asthma (*M* = 23.24, *SD* = 14.30) had significantly higher BDI scores than those without asthma (*M* = 19.31, *SD* = 13.32) *t* = −2.88, *p* = 0.004. There was still a significant effect of asthma on BDI scores after controlling for the effect of CTQ, *F*(1,473) = 6.16, *p* = 0.01, and BMI, *F*(1,500) = 7.29, *p* = 0.007, but not when controlling for the effect of TEI *F*(1,474) = 3.22, *p* = 0.07. Both the CTQ and TEI were positively related to BDI scores [CTQ: *r*(473) = 0.47, *p* < 0.001; TEI: *r*(474) = 0.43, *p* < 0.001], but BMI was not [BMI: *r*(500) = 0.07, *p* = 0.15].

### 3.3. Anterior insula reactivity to fearful faces among women with asthma and symptoms of PTSD, depression, and anxiety sensitivity

Among the neuroimaging sample, women with asthma did not show significantly higher PTSD symptoms [*t*(12.26) = −0.48, *p* = 0.64], depression symptoms [*t*(12.79) = 0.04, *p* = 0.97], or ASI scores [*t*(11.96) = 0.24, *p* = 0.82; Table 2] (Figures 1C-E).

**Figure 2:**
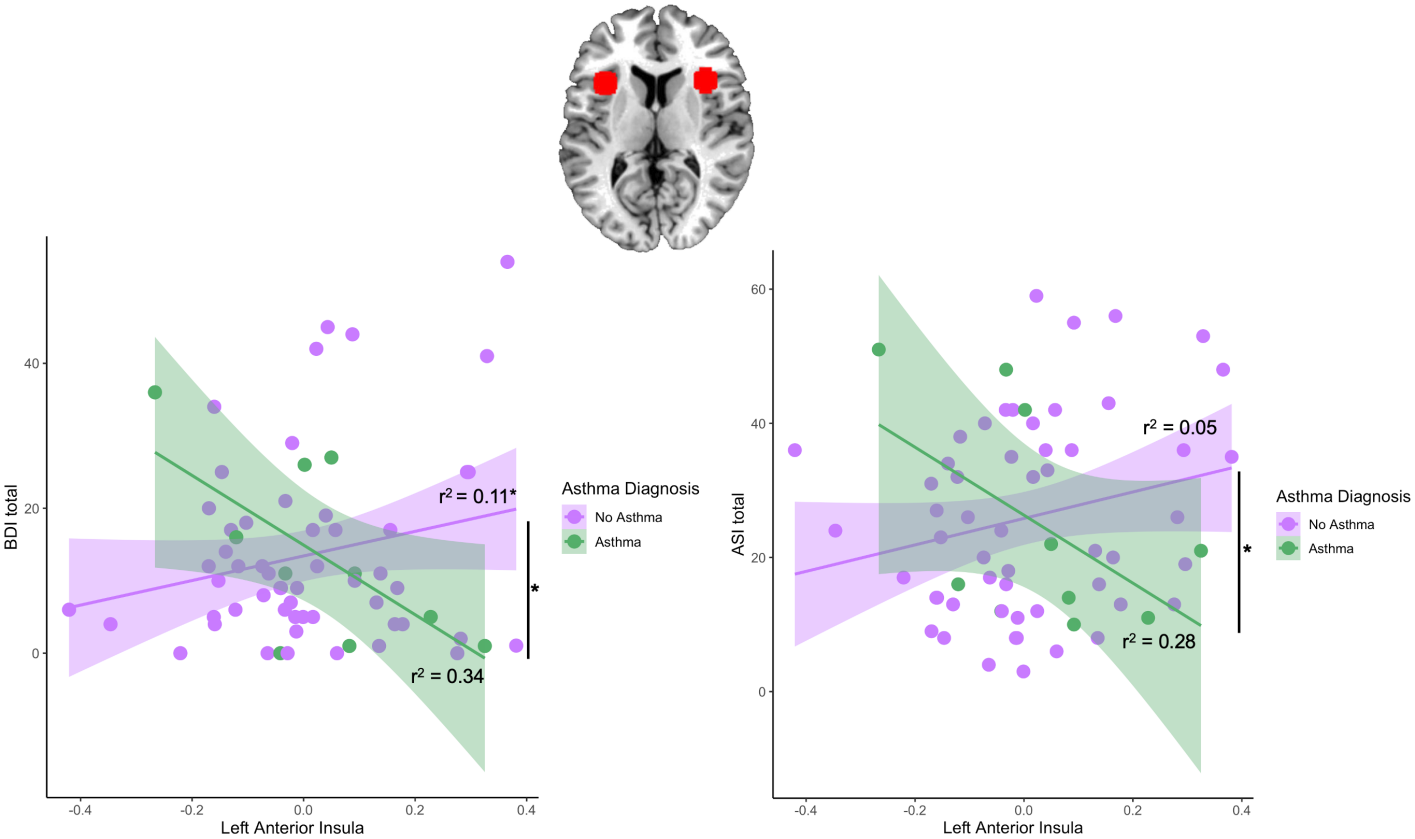
Region of interest and asthma-by-left anterior insula interaction effects predicting subsequent depression and anxiety symptoms. Figure depicts the right and left anterior insula region of interest (ROI). There was a significant interaction effect between left anterior insula and BDI scores and between left anterior insula and ASI scores. The no asthma group showed a significant positive correlation between the left anterior insula and BDI scores [*r*(52) = 0.33, *p* = 0.02] while the asthma group did not show a significant correlation [*r*(8) = −0.58, *p* = 0.08]. Both asthma [*r*(8) = −0.53, *p* = 0.11] and no asthma group [*r*(52) = 0.23, *p* = 0.10] did not show a significant correlation between the left anterior insula and ASI scores.

Regression models were used to examine the interaction effect between asthma*anterior insula reactivity. There was a significant interaction between asthma and left anterior insula reactivity (*β* = −63.59, *t* = −2.36, *p* = 0.02) but not right anterior insula reactivity in predicting depression symptom severity (*β* = −36.49, *t* = −0.92, *p* = 0.36; Table 3). Follow-up analyses revealed a significant positive correlation between the left anterior insula and depression symptoms in the no-asthma group [*r*(52) = 0.33, *p* = 0.02] while the asthma group did not show a significant correlation [*r*(8) = −0.58, *p* = 0.08; Figure 2]. Sensitivity analyses controlling for asthma medication and BMI revealed a significant interaction between asthma and left anterior insula reactivity in predicting depression symptom severity (*β* = −74.81, *t* = −2.47, *p* = 0.02). Similarly, for anxiety sensitivity, there was a significant interaction effect between asthma and left anterior insula reactivity (*β* = −73.13, *t* = −2.37, *p* = 0.02) but not right insula reactivity (*β* = −2.07, *t* = −0.05, *p* = 0.97; Table 3). Follow-up analyses revealed that neither the asthma [*r*(8) = −0.53, *p* = 0.11] nor no asthma group [*r*(52) = 0.23, *p* = 0.10] showed a significant correlation between the left anterior insula and ASI scores (Figure 2). Sensitivity analyses controlling for asthma medication and BMI revleaed a significant interaction between asthma and left anterior insula reactivity in predicting anxiety sensitivity symptom severity (*β* = −77.49, *t* = −2.09, *p* = 0.04). Finally, there was no significant interaction effect between asthma and left (*β* = −29.40, *t* = −1.16, *p* = 0.25) or right anterior insula reactivity (*β* = 21.33, *t* = 0.58, *p* = 0.56) for PTSD symptom severity (Table 3).

**Table 3:** Regression models using psychological measures and the anterior insula as the criterion.

	Estimate	Standard error	*T* value	Pr (>|*t*|)
PTSD symptom severity				
(Intercept)	12.65	5.48	2.31	0.02
Age	−0.02	0.13	−0.17	0.87
Asthma	2.80	4.17	0.67	0.50
Left Anterior Insula	2.25	9.56	0.24	0.82
Asthma*left anterior insula	−29.40	25.43	−1.16	0.25
(Intercept)	13.40	5.42	2.48	0.02
Age	−0.04	0.13	−0.33	0.74
Asthma	0.44	4.22	0.10	0.92
Right anterior insula	18.61	12.04	1.55	0.13
Asthma*right anterior insula	21.33	36.73	0.58	0.56
Depression symptom severity				
(Intercept)	17.32	5.80	2.99	0.00
Age	−0.10	0.14	−0.70	0.48
Asthma	1.13	4.41	0.26	0.80
Left anterior insula	17.17	10.11	1.70	0.09
Asthma*left anterior insula	−63.59	26.90	−2.36	0.02
(Intercept)	19.74	5.84	3.38	0.00
Age	−0.16	0.14	−1.14	0.26
Asthma	−0.30	4.55	−0.07	0.95
Right anterior insula	32.72	12.99	2.52	0.01
Asthma*right anterior insula	−36.49	39.61	−0.92	0.36
Anxiety sensitivity				
(Intercept)	18.13	6.65	2.73	0.01
Age	0.19	0.16	1.21	0.23
Asthma	1.20	5.06	0.24	0.81
Left anterior insula	19.31	11.61	1.66	0.10
Asthma*left anterior insula	−73.13	30.87	−2.37	0.02
(Intercept)	16.34	11.65	1.40	0.17
Age	0.15	0.17	0.90	0.37
Asthma	1.78	5.33	0.33	0.74
Right anterior insula	31.96	88.66	0.36	0.72
Asthma*right anterior insula	−2.07	46.34	−0.05	0.97

### 3.4. Effects of asthma on whole brain reactivity to fearful faces among women

There were no significant effects of asthma on the response to fearful>neutral faces on any brain region. Furthermore, an analysis of the interaction between asthma and PSS scores showed no significant results. We observed similar main effects of symptoms for the BDI, PSS, and ASI, with a general pattern of greater reactivity to fearful faces in right superior parietal cortex, right superior frontal gyrus, and right precuneus (Supplementary Figure S1).

## 4. Discussion

In the current study, we examined associations between trauma exposure, psychiatric disorders and symptoms, and asthma in a sample of women at high risk for repeated lifetime trauma. Our hypothesis on asthma diagnosis and trauma-related symptom severity was supported as women with asthma showed significantly greater PTSD and depression symptoms in the primary sample. These effects remained significant after controlling for childhood maltreatment but were no longer significant after controlling for the total number of different types of traumatic events experienced. In our neuroimaging group, we did not find support for our hypothesis relating higher insula reactivity to asthma and greater symptom severity in women. Instead, in the group without asthma, anterior insula reactivity to threat was linked with greater depression symptoms, while those with asthma showed the reverse pattern. This study is the first to examine anterior insula reactivity in relation to comorbid asthma and trauma-related psychopathology (PTSD, depression, and anxiety sensitivity).

Our work presents robust evidence for an association between asthma and PTSD and asthma and depression. Women with asthma in our primary sample were more likely to have greater PTSD severity and greater depression severity than those without asthma. This finding is consistent with prior literature. For example, one study in Europe found an association between trauma exposure and PTSD with airflow limitation and an obstructed respiratory system (Spitzer et al., 2011). Similarly, among female veterans in the US, those with PTSD had a 1.6-fold higher risk for asthma than those without PTSD (Dobie et al., 2004). For depression symptoms, the association with asthma appears to generalize across cultures, with data from the World Health Survey in 57 countries showing asthma to be significantly associated with depression (Loerbroks et al., 2012). Our study extends this association between asthma and PTSD and depression to a population of urban minority women in Atlanta. However, it should be noted that this association did not hold when controlling for trauma so it may be possible that trauma itself might be the major driver of an increase in asthma, with symptoms as the manifestation of trauma. This pattern did not hold up in our smaller neuroimaging sample of women, where we found no significant associations between asthma and PTSD, depression, or anxiety sensitivity. This could be due to the selective criteria used to screen women for the fMRI group (e.g., excluding for psychoactive medications, drug or alcohol misuse etc), as well as the low number of women with asthma vs. without asthma in the neuroimaging sample (n = 10; 15.63%) compared to the general sample (n = 161; 31.69%).

In looking closer at how anterior insula reactivity relates to asthma and symptom severity, we found that greater threat reactivity of the anterior insula was associated with greater depression symptoms in women without asthma. A different insula reactivity pattern was observed in women with asthma but larger studies of women with asthma are needed to confirm this, as the posthoc analysis in this group was non-significant due to low statistical power (n = 10). Anterior insula responses to threat cues were not directly associated with asthma or PTSD symptom severity. Our findings align with studies that found a positive relationship between depression severity and anterior insula reactivity. Studies have found greater anterior insula activity in depressed participants relative to controls including psychiatric inpatients with major depressive disorder, postmenopausal women, and unmedicated adolescents diagnosed with current depression (Wiebking et al., 2010; Perlman et al., 2012; McIntosh et al., 2022). There is limited research on the anterior insula and anxiety sensitivity, but our findings are also in line with prior studies that found greater activation in the left anterior insula among individuals with high anxiety sensitivity (Stein et al., 2007; Killgore et al., 2011; Yang et al., 2016). Our findings also align with prior neuroimaing studies that found increased insular reactivity in patients with PTSD and women exposed to domestic violence (Simmons et al., 2008; Bruce et al., 2013).

This study had a number of strengths. To date, it is the first study to examine insular cortex reactivity as a potential moderator of the link between asthma and PTSD, depression, and anxiety sensitivity. Prior research has established the co-occurrence of psychiatric disorders with inflammatory diseases, but limited knowledge is presented on the underpinnings and mechanisms behind this. Our work investigating associations between asthma and psychiatric symptom severity in relation to neuroanatomy allowed for a deeper exploration of the entanglement between somatic diseases and mental disorders. Secondly, urban minority women remain the most understudied population in research literature but are the most affected by psychiatric disorders and chronic lung diseases. This study examines the association between asthma and PTSD, depression, and anxiety sensitivity within a large, minority urban population allowing for more powerful effect sizes and direct, valuable investigations in populations most at risk.

Several limitations of the current study must be acknowledged when evaluating the results, in addition to what has been mentioned. Our participants were primarily Black or African American due to the makeup of the urban hospital where the data were obtained (Gillespie et al., 2009), and this could limit the generalizability of our findings. Our study also included only women. Future studies could benefit from including an equal proportion of both sexes, and to include men and women of various races and ethnicities. Furthermore, we did not have information on the severity and timing of asthma. Asthma is a variable condition classified by intermittent, persistent-mild, persistent-moderate, and persistent-severe subgroups (Colice, 2004). We were unable to see whether participants with asthma had experienced trauma before or after their diagnosis, and whether frequency of asthma attacks influences symptom severity scores. Additionally, information on smoking and nicotine usage was no collected due to the design of the parent study so we could not assess its effect on brain responsiveness between the groups. We also did not have information on the specifics of participant trauma history such as the timing of their traumatic events to be compared with asthma. To assess PTSD severity, this study used the PCL-5 for the general sample and the PSS for DSM-IV-TR for the neuroimaging sample, which had data collection beginning prior to DSM-5. It would be advantageous for future studies to use one standardized measure to assess PTSD and to use a clinician-administered interview as the assessment of PTSD. Our study also did not have anxiety sensitivity index scores collected for the primary sample as the parent study was not designed to investigate these variables specifically. Lastly, the cross-sectional and observational design of this study and complex nature of psychiatric disorders and asthma makes it difficult to establish a directionality between the two. Rather, our study provides information on the general relationship of asthma and traumatic stress in a community sample of women, not recruited from asthma clinics. The rates of asthma were determined by their natural incidence in this group, and we were therefore limited in our power to detect assocations with psychiatric symptoms, particularly in the neuroimaging study. Future studies may more accurately capture the relationship between asthma and PTSD, depression, and anxiety sensitivity symptoms by recruiting among patients being treated for asthma, and/or incorporating a longitudinal study design examining asthma rates and symptom severity over time.

In sum, our findings provided support for an association between asthma, and both PTSD and depression symptoms among trauma-exposed women. Moreover, the association between anterior insula reactivity and depression symptoms or anxiety sensitivity may depend upon whether the participant is experiencing an inflammatory health condition such as asthma. Stress, asthma, and psychiatric conditions remain an emerging and relevant area of research investigation that involves brain and body communication. Further understanding their comorbidity through neurobiological mechanisms and population differences is important in developing specialized, personalized treatments for individuals most at risk.

## Data availability statement

The data analyzed in this study is subject to the following licenses/restrictions: the data were collected beginning in 2005, and the informed consents for early years did not allow for public data sharing. Data can be requested upon inquiry to corresponding author. Requests to access these datasets should be directed to jennifer.stevens@emory.edu.

## Ethics statement

The studies involving humans were approved by Emory University Institutional Review Board and Grady Research Oversight Committee. The studies were conducted in accordance with the local legislation and institutional requirements. The participants provided their written informed consent to participate in this study.

## Author contributions

EL: Conceptualization, Formal analysis, Visualization, Writing – original draft. AR: Conceptualization, Data curation, Formal analysis, Supervision, Writing – review & editing. NF: Conceptualization, Investigation, Writing – review & editing. NM: Data curation, Writing – review & editing. CG: Investigation, Writing – review & editing. TE: Data curation, Formal analysis, Methodology, Writing – review & editing. BB: Writing- review & editing, Project administration, Funding acquisition, Resources. VM: Investigation, Project administration, Writing – review & editing. AP: Data curation, Investigation, Project administration, Writing – review & editing. TJ: Funding acquisition, Methodology, Project administration, Writing – review & editing. JS: Conceptualization, Data curation, Project administration, Resources, Supervision, Writing – review & editing.
