# Supplementary material for: Association between dimensions of trauma-related psychopathology and asthma in trauma-exposed women
Source: Front Behav Neurosci. 2023 Oct 31;17:1268877. doi: 10.3389/fnbeh.2023.1268877 (PMC10648896; doi:10.3389/fnbeh.2023.1268877)
Supplement: Supplementary file 1 [file Image_1.pdf]

Supplementary Results

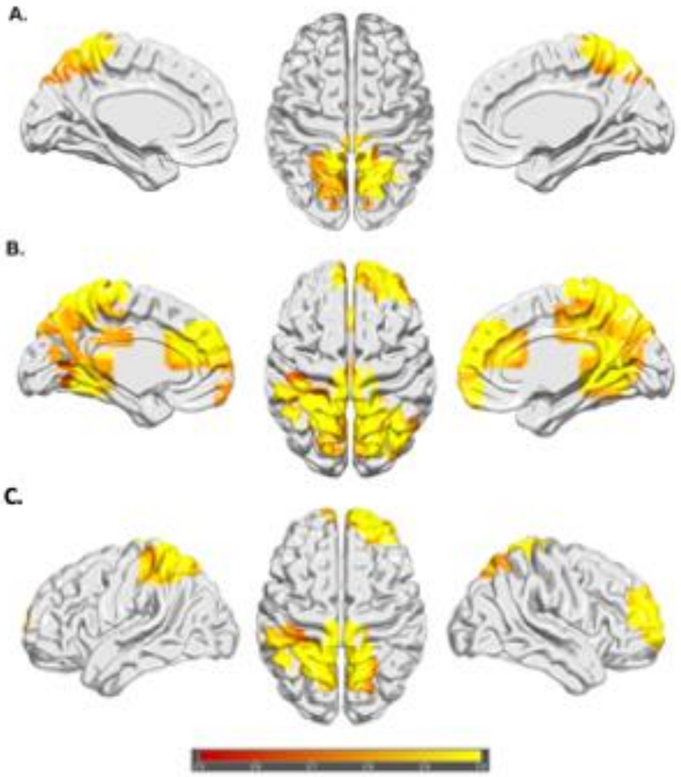

Supplementary Figure 1. Whole-brain clusters in response to fearful faces among individuals with different symptoms. There was a significant positive correlation between reactivity to fearful vs. neutral faces and (A) PSS scores in the right superior frontal gyrus (22, 68, 10; Z = 4.24 , k = 212) and right precuneus (6, -56, 66; Z= 3.74, k=212), (B) ASI scores in the right superior parietal gyrus (22, -64, 58, Z = 3.44, k = 125), (C) BDI scores in the right lingual gyrus (18, -48, 2; Z = 4.42, k = 219) and the right superior frontal gyrus (34, 60, 26; Z = 4.09, k = 219).
